# Use of a Neonatal-Mouse Model to Characterize Vaccines and Strategies for Overcoming the High Susceptibility and Severity of Pertussis in Early Life

**DOI:** 10.3389/fmicb.2020.00723

**Published:** 2020-04-17

**Authors:** Pablo Martin Aispuro, Nicolás Ambrosis, María Eugenia Zurita, María Emilia Gaillard, Daniela Bottero, Daniela Flavia Hozbor

**Affiliations:** Laboratorio VacSal, Instituto de Biotecnología y Biología Molecular (IBBM), Facultad de Ciencias Exactas, Universidad Nacional de La Plata, Centro Científico Tecnológico – Consejo Nacional de Investigaciones Científicas y Técnicas (CCT-CONICET), La Plata, Argentina

**Keywords:** pertussis, *Bordetella pertussis*, neonatal immunization, outer-membrane vesicles, protection

## Abstract

Newborns and unvaccinated infants, compared to other age groups, are more susceptible to pertussis infection, manifesting severe symptoms leading to a higher mortality. The recent increase in pertussis cases demands more effective strategies to overcome this major health problem. In parallel with maternal-immunization, neonatal-immunization (NI) is a strategy needing revision. Here, using the intranasal-challenge-mouse-model we evaluated the protective capacity of NI in both naïve-mice and those with maternally acquired immunity. We tested our acellular-vaccine-candidate based on outer-membrane-vesicles derived from *Bordetella pertussis* (OMVP) that induces Th2-profile but also the recommended Th-profile for protection: Th1/Th17-profile and CD4 T-memory-cells that reside in the lungs. Commercial acellular-vaccine (aP) and whole cell-vaccine (wP) inducing mainly Th2-profile and Th1-profile, respectively, were also tested. Analyzing the induced immunity and protection capability of NI included in 1- or 2-dose schedules with the same or different types of vaccine, we detected that the aP-vaccine administered in either single- or 2-dose schedules protected against sublethal *B. pertussis* infection. Schedules consisting of doses of aP neonatally and of OMVP or wP vaccine during infancy greatly reduced bacterial lung colonization while inducing the highest levels of high-avidity anti-pertussis toxin (PTx) IgG. That OMVP or wP neonatal dose did not interfere with the protection of transferred maternal immunity was especially encouraging. Moreover, OMVP- or wP used as a neonatal dose enhanced the quality of the humoral immune response in immunized pups. Antibodies generated by OMVP-or wP-vaccinated mice born to aP-immunized mothers were of higher avidity than those from mice that harbored only maternal immunity; but when mothers and neonates were immunized with the same aP-vaccine, the humoral response in the neonates was partially suppressed through the blunting of the level of anti-PTx IgG induced by the neonatal aP dose. These results demonstrated that neonatal immunization is a possible strategy to be considered to improve the current pertussis epidemiology. For neonates without maternal-immunity, mixed-vaccination schedules that include the aP- and OMVP-vaccines appear to be the most appropriate to induce protection in the pups. For offspring from immune mothers, to avoid blunting-effect, NI should be carried out with vaccines other than those applied during pregnancy.

## Introduction

The first month of life represents one of the most vulnerable periods for contracting infectious diseases that can be fatal to neonates. During 2018, 5.3 million children under 5 died and almost 50% of those fatalities occurred during the neonatal period before turning 1-month old (2.6 million newborn deaths, slightly 7.000 per day on average) (Liu et al., 2016; UNICEF, 2018). Some of the diseases that affect that age group are vaccine preventable, but the majority of the infants who die are not old enough to receive the number of vaccine doses required for inducing protection. Within this context, and in an effort to reverse this serious health problem, maternal immunization was implemented as a possible solution for various pathogens (Munoz et al., 2018). An example of that strategy is maternal immunization against pertussis or whooping cough. Pertussis is a vaccine-preventable respiratory disease mainly caused by the Gram-negative coccobacillus *Bordetella pertussis* that can affect all individuals regardless of age, but with the highest morbidity and mortality rates being among newborns and infants that have received either no vaccine or an incomplete vaccination schedule (Tanaka et al., 2003; Somerville et al., 2007; Masseria et al., 2016). Pertussis has resurged as a major public-health concern in many countries, including those with high vaccination coverages (Cherry, 2012; Tan et al., 2015). Until two decades ago, the control of the disease was mainly carried out through a vaccination scheme either with the traditional whole-cell vaccine (wP) or with the later developed acellular vaccine (aP) consisting of a three-dose primary series, with the first dose being administered as early as at 6 weeks of life and with subsequent doses being completed as of 6 months of age (World Health Organization [WHO], 2016). The weakness in the vaccines currently used lies in the duration of the induced immunity (shorter for aP-vaccinated individuals) together with the lack of optimal vaccination coverage. Furthermore, an evolution of the pathogen to greater vaccination resistance has contributed to the recent rise in the incidence of pertussis and fatalities (He and Mertsola, 2008; Klein et al., 2016; Eberhardt and Siegrist, 2017). While coverage has improved and better vaccines are designed, many countries have added vaccination boosters beyond the primary doses with the main aim being to reduce both the disease burden and the incidence in the most vulnerable populations. Maternal pertussis immunization during the third trimester of every pregnancy (27–36 weeks of gestation) is one of the recent strategies recommended in several countries to improve pertussis control in newborns and older infants (Healy, 2016; Hoang et al., 2016). The rationale for this strategy is that mothers are detected as the main source of infection for unprotected infants, who then are at high risk of complications and death, and that antibody-mediated immunity is achieved by placental transfer and breast-feeding (Cherry, 2016). In primate, pigs, and murine models and also in humans, results on the protection against pertussis in the neonates born to mothers that had been immunized during pregnancy have argued in favor of this strategy (Elahi et al., 2006, 2017; Warfel et al., 2014). Using a baboon model, Warfel et al. (2014) reported that maternal vaccination induced in newborns a full protection from severe pertussis symptoms following direct challenge with *B. pertussis* at few weeks of age. Using a porcine model, Elahi et al. (2017) demonstrated that immunization of pregnant sows with inactivated bacteria induced protection in piglets as a consequence of passive transfer of a wide range of cytokines in addition to pertussis-specific antibodies. Using mice, we detected that pertussis immunization of pregnant females with the acellular vaccine conferred protective immunity that is transferred both transplacentally and via offspring breast-feeding without compromising the protection boostered by subsequent infant vaccination (Gaillard et al., 2017). In humans, extremely significant data recently obtained would support the following statements: (1) human maternal immunization is safe for women and fetuses, (2) maternal-immunization strategy is able to decrease the lethality rates in newborns and infants born to immunized mothers (Fabricius et al., 2018; Gentile et al., 2018) and (3) maternal immunization is highly protective against pertussis, especially in the first 2 months of life, but also up to the first year. Moreover, Baxter et al. (2017) found that maternal immunization provided additional protection for infants who received the first dose of pertussis vaccine.

Unfortunately, vaccination coverage in pregnant women for pertussis and other pathologies, although improved over time, is still inadequate, leaving mothers and newborns without specific immunity against pertussis. The situation is further complicated for premature neonates because even if the mother is immunized, her immunity may not be transferred to the newborn, as its birth was premature. Within this context, whereas approaches to increase maternal-immunization coverages are being improved and evidence has indicated that maternal immunization can be performed earlier, in the second trimester of pregnancy; neonatal immunization could become a complementary strategy to improve pertussis epidemiology in newborns. Neonatal immunization is an extremely attractive approach because, in terms of coverage, high levels could be achieved since birth is a reliable point of contact with medical care. Moreover, neonatal vaccination would represent an opportunity for the protection of premature newborns (Kent et al., 2016). In fact, vaccines like Bacillus Calmette Guérin (BCG) and those for hepatitis B (HBV) and polio have proven to be safe and effective at birth despite the difference in immunity and the reduced immune responses detected in the vaccinated newborns. In the example of pertussis, evidence has been cited of successful neonatal immunization both in animal models (Roduit et al., 2002; Warfel et al., 2014) and in humans (Wood et al., 2010), thus pointing to the feasibility of that vaccination strategy. Nevertheless, whether this approach will interfere with future vaccinations during infancy or with transferred maternal antibodies still remains controversial. In fact, certain studies suggested that, in infants of mothers vaccinated during pregnancy, the circulating maternal pertussis antibodies blunt the specific antibody responses in the neonate after the administration of aP vaccines in a primary series (Ladhani et al., 2015; Vono et al., 2019). In addition, certain evidence suggests that a dose of aP at birth may suppress the immune response to subsequent aP doses and suppress the immune responses to vaccines against *Haemophilus* influenza type B and hepatitis B virus (Knuf et al., 2008); but that interaction seems to depend on the brand of aP vaccine. What is of utmost relevance here is that the majority of the reported data were obtained by using the current acellular vaccines. Those vaccines were developed during a later period in response to reports on the induction of adverse reactions associated with the vaccinations involving the first pertussis vaccine developed, which preparation consisted in a suspension of killed *B. pertussis* bacteria (wP) that induced a potent antibody and Th2-polarized response plus weak Th17 responses (Ross et al., 2013; Brummelman et al., 2015). Unfortunately, immunization with the aP vaccines has appeared to be less effective than the current wP vaccines in inducing immunologic memory (including tissue resident T cells, Trm) and in conferring long-term protection against pertussis (Brummelman et al., 2015; Allen et al., 2018; Borkner et al., 2018).

We have designed a new multi-antigen acellular pertussis-vaccine formulation obtained from membrane components of *B. pertussis* (outer-membrane vesicles, OMVs) that shares the beneficial properties of the current aP vaccines in terms of biosafety and those of the wP vaccines with respect to immunogenicity and protective capability (with the international patent having been granted in the United States and in process in other countries, Application Number: PCT/IB2014/060143) (Roberts et al., 2008; Asensio et al., 2011; Gaillard et al., 2014). We have reported that the OMV-based vaccine (OMVP) was capable of inducing a more robust immune response than current aP vaccines with a Th1/Th17 and Th2 cellular profile (Zurita et al., 2016) that confers long lasting protection against *B. pertussis* (Gaillard et al., 2014). Thus, in this study we used the mouse model and different types of vaccines and vaccination schedules in order to substantially enhance our understanding of the efficacy of neonatal pertussis immunization in the protection of offspring that have been born to vaccinated and unvaccinated mothers. We also determined the potential interference of vaccination during the neonatal period with the eventual protection achieved by the primary vaccination against *B. pertussis.*

## Materials and Methods

### Mice

BALB/c mice (4 weeks old), obtained from the Facultad de Ciencias Veterinarias of the Universidad Nacional de La Plata, were kept in ventilated cages and housed under standardized conditions with regulated daylight, humidity, and temperature. The animals received food and water *ad libitum*. Day 1 of gestation was determined when vaginal plugs were observed. Breeding cages were checked daily for new births, and the pups were kept with their mothers until weaning at the age of 3 weeks.

The animal experiments were authorized by the Ethical Committee for Animal Experiments of the Faculty of Science at La Plata National University (approval number 004-06-15, 003-06-15 extended in validity until August 10, 2023).

### *Bordetella pertussis* Strain and Growth Conditions

*Bordetella pertussis* Tohama phase I strain CIP 8132 was used throughout this study as the strain for challenge in the murine model of protection. *B. pertussis* was grown in Bordet–Gengou agar supplemented with 15% (v/v) defibrinated sheep blood (BG-blood agar) for 72 h at 36.5°C. Isolated colonies were replated in the same medium for 24 h and then resuspended in phosphate-buffered saline (PBS: 123 mM NaCl, 22.2 mM Na2HPO4, 5.6 mM KH2PO4 in MilliQ^®^ nanopure water; pH 7.4). The optical density at 650 nm was measured and serial 10-fold dilutions plated onto BG-blood agar to determine the density of the challenge inoculum.

### Vaccines

The maternal immunization protocols were performed with the three-valent pertussis aP BOOSTRIX^®^ (GSK, GlaxoSmithKline), with the composition per human dose: pertussis toxoid (8 μg), pertactin (2.5 μg), filamentous hemagglutinin (8 μg), tetanic toxoid (20 IU), and diphtheria toxoid (2 IU). For all experiments, the immunization was carried out through the use of a 1/10 human dose of that vaccine, hereafter referred to as a mouse dose. The vaccinations of neonatal or infant mice were performed with 1 mouse of the aP, a commercial wP vaccine (DTP vaccine, PT. BIO FARMA, Indonesia), or the *B. pertussis*–outer-membrane-vesicle–based vaccine formulated by us as previously described (Asensio et al., 2011), to be referred to as the OMVP vaccine.

### Experimental Protocol

#### Maternal, Neonatal, and Infant Immunization

Female BALB/c mice (*n* = 8) were immunized (i.m) with three doses of commercial acellular vaccine (aP) Boostrix ^TM^ at 1/10 of the human dose (HD) at days 0 and 14. This dose was selected based on the basis of our previous work and also since, in the mouse-intranasal-challenge model, 1/10 HD had appeared to be more sensitive for detecting variations in the efficacy of the different vaccines than 1/4 HD (Queenan et al., 2014). Before applying the third dose of the vaccine, the females were housed with males within the same cage and checked daily for pregnancy. When mucosal vaginal plug was detected, a third vaccine dose was applied. Pregnancy eventually occurred after detection of the vaginal mucosal plug. Mouse couples stayed cohoused until the end of the experiment. Non-immunized mice were used as a negative control of protection. Offspring born to either immunized or non-immunized mothers were immunized subcutaneously in the upper back with 1/10 the human dose of aP, wP, or OMVP [50 μl at the concentration used by Asensio et al. (2011)] at the age of 1 week (neonates) and/or 3 weeks (infants). The ages of the mice used for the neonatal and infant immunizations were selected on the basis of published antibody responses to vaccine antigens and the colonization of secondary lymphoid organs where 7-day-old mice had been reported to be similar to newborn children (Adkins, 2005). Immunologic maturity is thereafter acquired gradually over the next 3 to 5 weeks of life. These schedules had been previously used to investigate the immune responses induced in a neonatal-mouse model by heterologous prime boosting, with the live attenuated pertussis vaccine BPZE1 (a derivative of the *B. pertussis* strain Tohama I) being used for priming and aPV for boosting (Feunou et al., 2014). Those authors had compared the immune responses and protection conferred in animals primed as neonates (at 7 to 10 days old) with those primed in infancy (at 3 weeks old) (Feunou et al., 2014).

To evaluate the protective capability induced by the different vaccination strategies used, we performed an intranasal challenge with a sublethal dose [10^6^–10^7^ colony-forming units (CFUs) in 40 μl^–1^] of *B. pertussis* Tohama phase I at 14 days after the last immunization. Seven days after the challenge, the mice were sacrificed, and their lungs were harvested, homogenized in PBS and plated in serial dilutions onto BG-blood agar to count the colonies formed after incubation at 36.5°C for 3–4 days. At least three independent assays were performed.

#### Enzyme-Linked Immunosorbent Assay (ELISA)

As we described previously (Bottero et al., 2016), plates (Nunc A/S, Roskilde, Denmark) were coated with the purified pertussis toxin (PTx) at 3 μg/ml in 0.5M carbonate buffer pH 9.5, by means of an overnight incubation at 4°C. The rinsed plates were then blocked with 3% milk in PBS (2 h, 37°C) before incubation with serially diluted samples of mouse serum (1 h, 37°C). In the experiments described above, blood samples were collected from the mothers at delivery, from the pups at weaning, from the mothers and the pups before pup challenge, and from the pups 13 days after immunization. The sera were obtained after leaving the blood samples to clot for 1 h at 37°C followed by centrifuging for 10 min at 7,000 × *g*. The IgGs from individual or pooled sera bound to the plates were detected after a 2-h incubation with horseradish-peroxidase–labeled goat anti-mouse IgG (Invitrogen, United States) at 1:8,000. For measuring the IgG isotypes, the bound antibody was incubated with horseradish-peroxidase labeled subclass-specific anti(mouse IgG1) at 1:8,000 or anti(mouse IgG2a) at 1:1,000 (Sigma, Aldrich). The substrate used was 1.0 mg/ml *o*-phenylendiamine (Bio Basic Canada, Inc.) in 0.1M citrate-phosphate buffer, pH 5.0 containing 0.1% (v/v) hydrogen peroxide. Optical densities were measured with Titertek Multiskan Model 340 microplate reader (ICN, United States) at 492 nm and then plotted as a function of the log of the (serum dilution)^–1^. A successful assay for each antibody sample produced a sigmoidal curve in this type of plot. The titer of each antibody sample was determined from this curve by identifying by GraphPad Prism^®^ software the concentration (expressed as inverse of the dilution of the antibody) that provokes a half way between the basal response and the maximal response. The cut-off levels determined for IgG, IgG1, and IgG2a assays were 12.5 ± 3.6, 1.9 ± 0.9, and 5.8 ± 2.7, respectively.

From the experimental protocol, performed in triplicate, one representative experiment is presented throughout the Results.

#### Avidity Assay

Avidity was measured by an ELISA-elution assay as the overall strength of binding between antibody and antigen, using plates incubated for 15 min with increasing concentrations of ammonium thiocyanate (NH_4_SCN) from 0 to 0.375M. Antibody avidity was defined as the amount (percentage) of antibody eluted for each NH_4_SCN concentration.

#### Protein Assay

The protein content was estimated by the Bradford method with BSA as the standard (Bradford, 1976).

#### Sodium Dodecylsulfate-Polyacrylamide Gel Electrophoresis (SDS-PAGE) and Immunoblotting

Purified PTx was solubilized in Laemmli sample buffer (Laemmli, 1970) and heated at 100°C for 10 min. The purified protein was separated by SDS-PAGE and then transferred onto polyvinylidine difluoride (PVDF; Immobilon P, Millipore) at constant voltage (100 V). After the transfer, the PVDF membranes were probed with immune sera or non-immune sera (1: 1,000) followed by incubation with anti(mouse IgG) conjugated with alkaline phosphatase at a 1:1,000 dilution. Nitroblue tetrazolium and 5-bromo-4-chloro-3-indolyl-phosphate were used as the phosphatase substrates according to the manufacturer’s protocol (Biodynamics SRL Buenos Aires Argentina).

### Statistical Analysis

The data were evaluated statistically by a one-way analysis of variance (ANOVA) followed by the Tukey test *post hoc* (via the GraphPad Prism^®^ software). Differences were considered significant at a *p* < 0.05.

## Results

### Neonatal Immunization and Protection Against *B. pertussis* Infection in Naïve Mice

**FIGURE 1:**
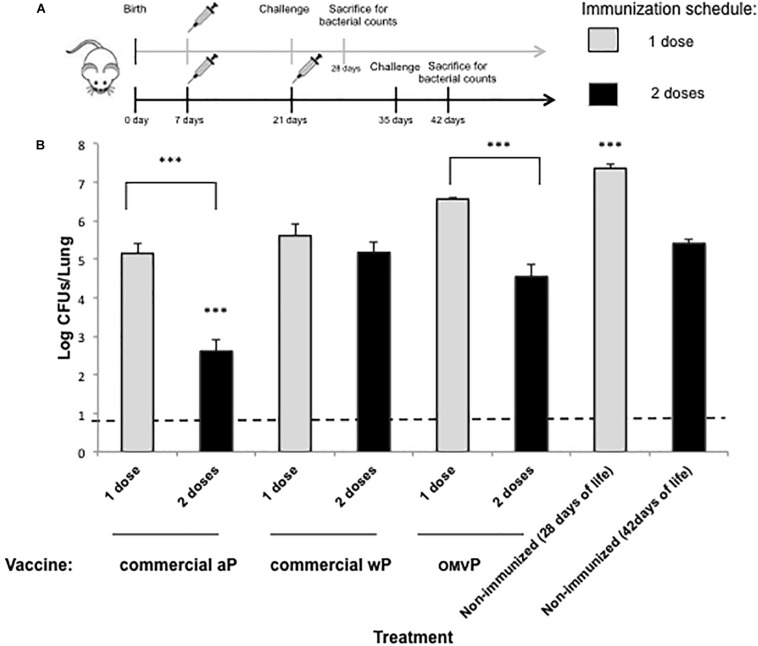
Effect of neonatal immunization on the protection of offspring against *Bordetella pertussis* infection. **(A)** Schematic representation of vaccination and challenge protocols. Seven-day-old neonatal mice were vaccinated with a commercial aP, OMVP or a commercial wP vaccines (*n* = 8 in each group). For the two-dose vaccination schedules consisting in two doses, the second dose (black horizontal arrow) was administered 14 days after the first dose. Mice immunized with 1 dose (gray horizontal arrow) or 2 doses (black arrow) were challenged with *B. pertussis* at 28 and 35 days after birth, respectively. Non-immunized mice of the same age (used as a negative control for protection) were also challenged with *B. pertussis*. **(B)** Protection of offspring through the vaccination schedules of **(A)**. The number of bacteria recovered from the mouse lungs, expressed as the log_10_(CFUs per lung), is plotted on the *ordinate* for the type and vaccine schedule indicated on the *abscissa*, with the data representing the means ± the SD. The dotted horizontal line demarcates the lower limit of detection. ^∗∗∗^*p* < 0.001 for both the non-immunized mice versus the 1-dose–immunized mice and the aP-immunized mice versus all the other treatments performed for the 2-dose–schedule assays.

**FIGURE 2:**
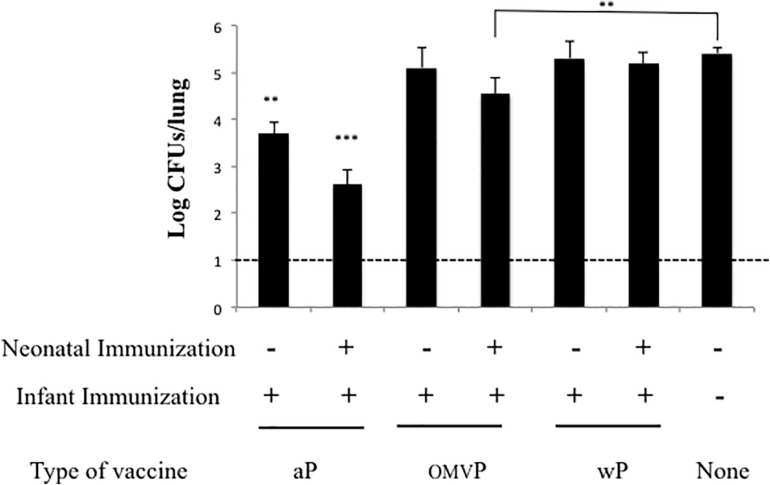
Effect of neonatal immunization on the first dose administered during the infancy. The infant doses were administered in both immunized or non-immunized mice at 21 days during the neonatal period in either the presence or the absence of a neonatal dose at 7 days of age. In the neonates immunized (upper indications on the *abscissa*), the type of vaccine used was the same as that administered during infancy (lower indications on the *abscissa*). The non-immunized mice were used as a negative control of protection. All the groups of mice (*n* = 8) were challenged with *B. pertussis* at 35 days after birth. The number of bacteria recovered from mouse lungs, expressed as the log_10_(CFUs per lung), is plotted on the *ordinate* for the type and vaccine schedule indicated on the *abscissa*, with the data representing the means ± the SD. The dotted horizontal line indicates the lower limit of detection. ^∗∗∗^*p* < 0.001, ^∗∗^*p* < 0.05.

To evaluate the protection of naïve offspring against *B. pertussis* infection through neonatal-vaccine–induced immunity, the model we used involved the immunization of neonatal mice followed by a sublethal intranasal challenge with *B. pertussis*. Neonatal (7-day-old) BALB/c mice were either simply primed or primed and then boosted subcutaneously 2 weeks later (at 21 days-old; Figure 1A) with the following vaccines selected for this study: commercial aP, which induces mainly a Th2 profile, wP, which generates mainly Th1 and Trm response; or OMVP vaccine; which induces a mixed Th1/Th17 and Th2 mixed profile plus Trm response (Zurita et al., 2019). Non-immunized mice sacrificed at 28 or 42 days of age served as the negative controls for 1- and 2-dose-vaccination schedules, respectively. For both vaccination schedules the mice tested were challenged (intranasally) with 106–107 CFUs of *B. pertussis* Tohama phase I at 14 days after the last immunization (Figure 1). Significant differences were observed between the bacterial counts recovered from the lungs of mice immunized with 1 dose of vaccine and those from the negative control group (Figure 1B, gray columns; *p* < 0.001) were observed. The highest protection was detected in the animals immunized during the neonatal period with aP (with the CFUs being reduced by some 2 orders of magnitude in comparison to the non-immunized mice; *p* < 0.001). This result is in accordance with the information on the neonatal immune system indicating a preferential differentiation of the CD4^+^ helper T cells into Th2 cells in response to danger signals and antigens (Mohr and Siegrist, 2016) along with the induction of mainly a Th2 profile by the aP vaccine. Experiments involving the 2-dose–vaccination schedule in which one dose was administered at 7 days of age and the second at infancy (21 days of age) in comparison with schedules that included a single dose at infancy indicated that neonatal vaccination did not interfere with the eventual protection induced by the primary vaccination (Figure 2). Moreover, while a single neonatal dose with any of the vaccines tested induced protection (Figure 1B, gray columns), a single dose in infancy did not confer protection unless the vaccine used for the immunization was the aP (Figure 2, *p* < 0.05). In addition, with the aP vaccine, a higher protection was detected in the 2-dose schedule compared to either a single neonatal aP dose (difference of 2.25 orders of magnitude, *p* < 0.001, Figure 1) or to the 2-dose schedule performed with the other 2 vaccines tested here (difference of at least two orders of, magnitude *p* < 0.001; Figure 2). For the other vaccination schedules tested: wP-wP or OMVP-OMVP, non-significant differences were detected compared to the single-vaccination schedule during the neonatal period (Figure 1B, black columns vs. gray columns).

### Effect of Immunization Schedules Involving Two Different Types of Vaccines on Protection Against *B. pertussis* Infection in the Neonatal-Mouse Model

**FIGURE 3:**
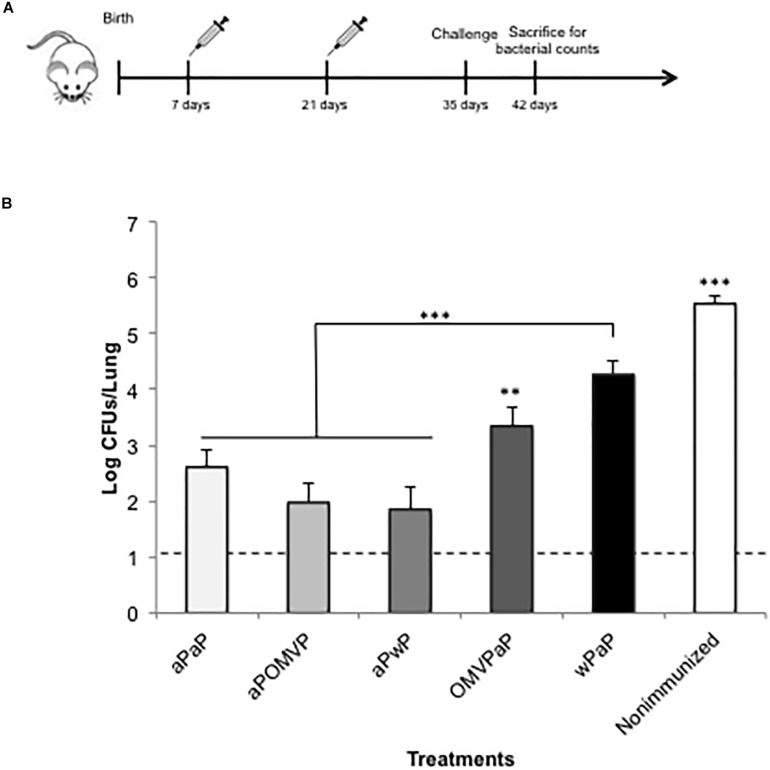
Effect of 2-dose schedules including neonatal immunization on protection of offspring against *B. pertussis* infection. **(A)** Schematic representation of vaccination and challenge protocols. Seven-day-old neonatal mice were vaccinated with a commercial aP, OMVP, or a commercial wP vaccine (*n* = 8 in each group). The second dose, involving a different type of vaccine from the first, was administered 14 days after the first dose. Non-immunized and aP-aP–immunized mice were used as respective negative and positive controls of protection. All the mice were then challenged with *B. pertussis* at 35 days after birth followed by sacrifice at 42 days. **(B)** Protection of offspring through the vaccination schedules of **(A)**. The number of bacteria recovered from mouse lungs, expressed as the log_10_ of the CFUs per lung, is plotted on the *ordinate* for the combination of vaccine types indicated on the *abscissa*, with the data representing the means ± the SD. The dotted horizontal line demarcates the lower limit of detection. ^∗∗∗^*p* < 0.001, ^∗∗^*p* < 0.05.

On the basis of the results indicating that the inclusion of aP vaccine in the neonatal immunization model exhibited the highest protective capability, we decided to evaluate whether adding aP to the 2-dose-vaccination schedule that contained a second type of vaccine was able to induce higher levels of protection. To that end, we performed the *in vivo* protection assays described above using the following schedules that included aP as the primary or the booster dose—that is: aP-OMVP; OMVP-aP; aP-wP, and wP-aP. As a control for protection, we included a group of mice vaccinated with the schedule that up to now conferred the highest level of protection that is the aP-aP schedule. As a negative control, we used non-immunized mice. Of interest to us was that we observed the highest levels of protection in those schedules in which the neonatal dose was performed with the aP vaccine (Figure 3). The reduction in the CFUs recovered from lungs of the aP- aP-, aP- OMVP-, and aP-wP–immunized mice ranged from 2.9 to 3.6 orders of magnitude (non-significant differences between those treatments) in comparison to the highly significant difference relative to non-immunized animals (*p* < 0.001). Indeed, the protection levels conferred by OMVP-aP or wP-aP (Figure 3) were higher than those detected with the OMVP-OMVP or wP –wP schedules (Figure 1B). The comparison of theses results is valid because in both sets of experiments we used for the challenge the same sublethal dose of *B. pertussis.*

### Anti-Ptx Antibodies Induced by the 2-Dose Vaccination Schedules

**FIGURE 4:**
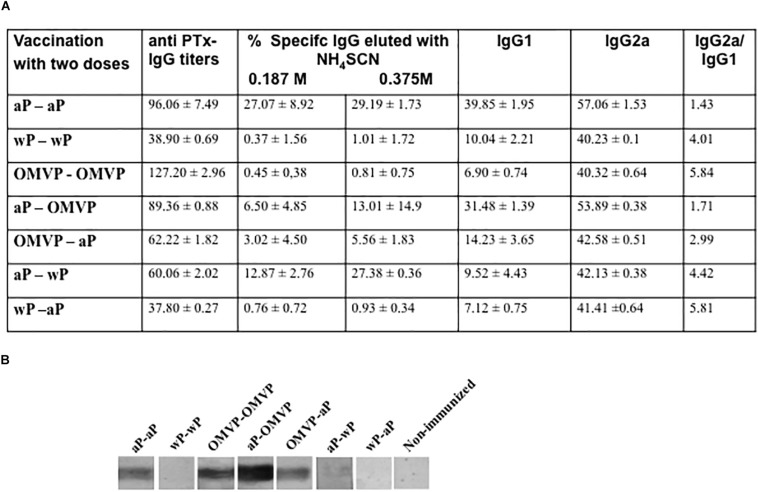
Anti-Ptx antibodies induced by 2-dose vaccination schedules. **(A)** Anti-PTx IgG titers along with the IgG isotypes were measured 14 days after the second vaccination dose. The titers are expressed as the geometric mean of the data from each group. The cut-off levels for IgG, IgG1, and IgG2a assays were 12.5 ± 3.6, 1.9 ± 0.9, and 5.8 ± 2.7, respectively. No values were detected in the non-immunized control animals. The values detected were significantly different from those of the non-immunized mice (*p* < 0.05). The avidity of IgG antibodies was also measured by ammonium thiocyanate (NH_4_SCH) elution 14 days after the second dose and is expressed as the percentage of PTx-specific antibodies eluted. **(B)** Immunoblotting of purified PTx separated by 12.5% (w/v) SDS-PAGE and probed with polyclonal sera obtained from immunized mice. The sera are designated according to the vaccination schedule used to raise the immune response in the donor mice.

In this study, the IgG titers against pertussis toxin (anti-PTx IgG) were measured regularly after the last immunization. None or very low values of those titers were detected in a single-dose-vaccination schedule (data not shown). These results on a single neonatal dose were in agreement with those reported by Vono et al. (2018) for influenza vaccines. For the 2-dose-vaccination schedules, the PTx-specific antibody responses were detected in all the immunized mice (Figure 4A). Among the schedules that included the same type of vaccine for the first and second dose, the aP-aP and OMVP-OMVP vaccination schedules induced the highest values of anti-PTx IgG titers (Figure 4A). High values of the IgG titers were also detected in those mixed schedules that included the aP vaccine in the neonatal immunization. An interesting observation was the higher proportion of anti-PTx IgG with high avidity detected in the sera of the mice immunized with schedules that contained a neonatal dose of OMVP or wP in comparison to those induced in mice immunized with schedules started with an aP neonatal dose (Figure 4A). Consistent with the anti-PTx IgG titers and avidity results, a differential PTx-recognition intensity was observed in blots from SDS-PAGE gels probed with the vaccine-induced sera that were tested (Figure 4B). Thus, Figure 4 illustrates how, with the heterologous schedules involving different types of vaccine for neonatal and infant immunizations, the recognition of PTx was more intense in those that began with an aP neonatal dose. For the mixed schemes in which aP was used as a dose in infancy, the recognition signal for PTx was either reduced (for the OMVP-aP schedule) or null (for the wP-aP schedule) compared to the signals detected when the aP vaccine was used as the neonatal dose. All these results pointed to the occurrence of a prime-boost effect.

For the 2-dose schedules with the same vaccine type in both doses, the recognition of PTx was sharp in those combinations in which the vaccine induced mainly a Th2 profile, either aP or OMVP. By contrast, for a 2-dose schedule with a vaccine that induced a robust Th1 profile response, such as with the wP vaccine, the PTx detection was null.

Another important observation was that the schedules that included OMVP or wP triggered murine-antibody responses with an IgG2a/IgG1 > 1.5 (Figure 4A). The high IgG2a/IgG1 ratio observed in the schedules consisting of a neonatal dose with OMVP or wP and an aP-dose in infancy not only suggested that OMVP or wP skewed the immune response toward a Th1 profile but also provided evidence of a heterologous prime-boost effect.

### Neonatal Immunization and Protection Against *B. pertussis* Infection in Mice With Maternal Immunity

**FIGURE 5:**
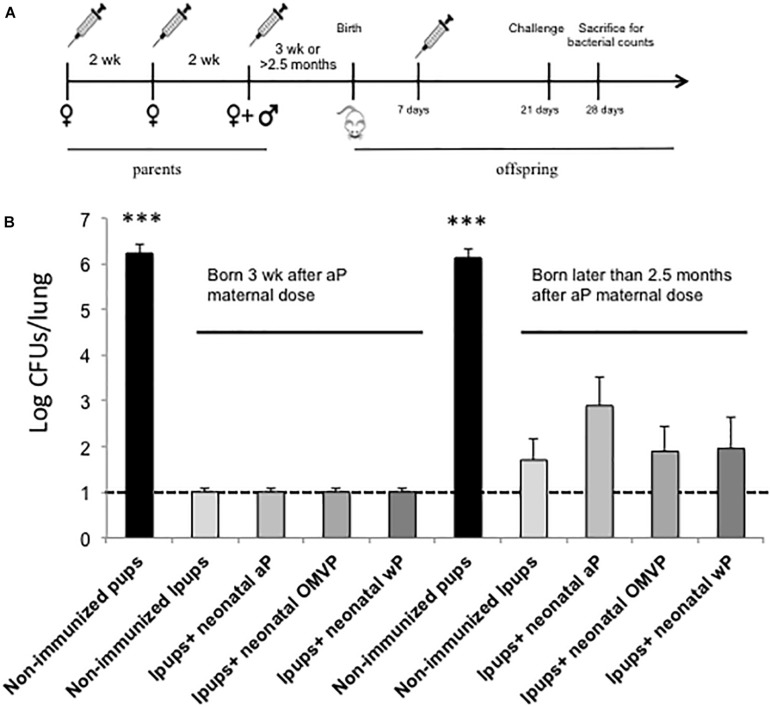
Effect of neonatal immunization on the protection of pups with maternal immunity against *B. pertussis* infection. **(A)** Schematic representation of the vaccination and challenge protocols. Seven-day-old neonatal mice born either 3 weeks or later than 2.5 months after maternal aP immunization were vaccinated with a commercial aP or wP or the OMVP vaccine (*n* = 6 in each group). Non-immunized mice were used as a negative control for protection. All the groups were challenged with *B. pertussis* at 28 days after birth. **(B)** Protection of offspring through the vaccination schedules of **(A)**. The number of bacteria recovered from the mouse lungs, expressed as the log_10_ of the CFUs per lung, is plotted on the *ordinate* for the treatments indicated on the *abscissa*, with the data representing the means ± the SD. The dotted horizontal line indicates the lower limit of detection. The left side of the panel depicts the CFU values for the different groups of mice born 3 weeks after the last maternal vaccination, while the right side contains the corresponding data for the same experimental groups born 2.5 months after that last maternal vaccination. ^∗∗∗^*p* < 0.001 non-immunized mice versus immunized Ipups. A significant difference (*p* < 0.05) was also recorded between the CFU values detected in the aP-vaccinated Ipups born 2.5 months after maternal immunization and those values detected in the non-vaccinated and vaccinated Ipups born 3 weeks after the completion of maternal immunization.

To evaluate the effect of neonatal immunization in mice with maternal immunity (hereafter referred to as Ipups), we first had to generate a pool of adult female mice primed by anti-pertussis vaccination. Since in humans the aP vaccine is used for maternal immunization, we included this type of vaccine for the female-vaccination schedule in our experiments. We accordingly vaccinated adult female mice with 1/10 of the human dose of aP in three doses with the third one being administered during pregnancy (Gaillard et al., 2017). By using this model in a previous study, we demonstrated that infants born to vaccinated mothers were protected against *B. pertussis* infection, with a humoral immune response being mainly responsible for the protection detected in the Ipups. In the present experiments, we assessed whether the neonatal immunization could be used in mice with maternal immunity. To that end, mice born to vaccinated mothers were immunized at 7 days of age with each one of the three tested vaccines: the commercial aP and wP vaccines or our novel OMVP vaccine candidate (Figure 5A), with non-immunized mice being used as a control group.

To evaluate the protection, all the mice were challenged at 21 days of age with 10^6^ CFUs of the *B. pertussis* strain Tohama phase I. As we described before (Gaillard et al., 2017), all the unvaccinated animals born to immunized mothers were protected against *B. pertussis* infection. The CFU counts in the lungs of those Ipups, regardless of whether or not they had received a vaccine dose during the neonatal period, were not detectable. Moreover, the levels of protection were equally high independently of the type of vaccine used as a neonatal dose (Figure 5B). These results were obtained in mice born 3 weeks after their mothers received the last aP dose during pregnancy. An important degree of protection against *B. pertussis* infection was also observed in mice born later than 2.5 months after their mothers had received the last dose during pregnancy (Figure 5B). The levels of CFUs detected in this instance, however, were slightly higher than those recorded in infants born 3 weeks after the mothers had received their final dose during pregnancy. In particular, the highest values of CFUs recovered were detected in those animals that were vaccinated with aP vaccine (with CFU counts approximately 10-fold higher than those of the non-vaccinated Ipups, *p* < 0.05), thus indicating that neonatal immunization with aP interfered with the functionality of the maternal antibodies (Figure 5B).

**FIGURE 6:**
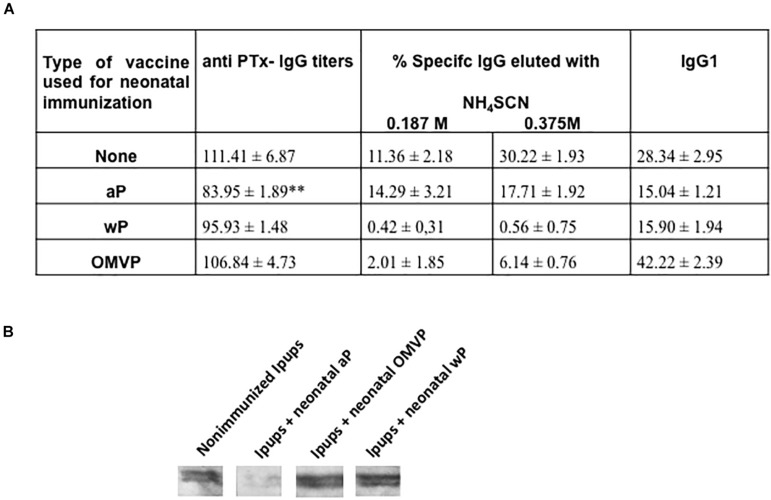
Anti-Ptx antibodies in pups born to aP immunized mothers. **(A)** Anti-PTx IgG titers and the IgG1 isotype were measured at 21 days of life in the Ipups. The titers are expressed as the geometric mean of the data from each group. No values were detected in pups born to non-immunized mothers used as the control group (not indicated in the figure). The values detected in the aP-immunized Ipups were significantly lower (*p* < 0.05) than those of the non-immunized mice. The cut-off levels for IgG and IgG1 assays were 12.5 ± 3.6 and 1.99 ± 0.98, respectively. The avidity of the IgG antibodies was also measured in the Ipups at 21 days of life, as indicated by the percentages of PTx-specific antibodies eluted after treatment with increasing concentrations of ammonium thiocyanate (NH_4_SCN). **(B)** Immunoblotting of purified PTx separated by 12.5% (w/v) SDS-PAGE and probed with the polyclonal antisera obtained from immunized mice. The sera are designated according to the vaccination schedule used to raise the immune response in mice.

As to the antibody titers, we quantified of anti-PTx IgG antibodies in the vaccinated pups. In contrast to the undetectable titers in the pups born to non-immunized females (data not shown), significant levels of anti-PTx IgG were present in the sera of the Ipups (Figure 6A). The lowest values of IgG titers were detected in the aP-immunized neonates (*p* < 0.05, vs. all other treatments). Non-significant differences were detected between the IgG levels of wP or OMVP vs. non-immunized Ipups animals (Figure 6A). Since one of the most transferred IgG isotypes from the mother to the fetus is IgG1, we also evaluated this isotype in vaccinated or unvaccinated Ipups (Figure 6A). The results indicated again that the Ipups immunized during the neonatal period with aP manifested IgG1 titers significantly lower than those in the non-immunized Ipups (*p* < 0.05).

As an indication of the antibody avidities, we observed that in mice with maternal aP-induced immunity that were also immunized with aP the percentages of elution with NH_4_SCN were considerably higher than those detected in the sera of Ipups immunized with wP or OMVP (Figure 6A).

Consistent with the ELISA-IgG and avidity results obtained, we observed a marked intensity in the recognition of PTx in blots from SDS-PAGE gels probed with some of the tested vaccine-induced sera (Figure 6B). We noted that, although with sera from both the non-immunized and immunized Ipups with OMVP- or wP, the PTx band was clearly recognized by the specific antibodies, with the sera from Ipups that received aP as a neonatal dose that recognition against PTx was null.

The data presented corresponded to those obtained in mice born 3 weeks after their mothers were immunized during pregnancy. Similar results were observed in the pups born to mothers whose immunization during pregnancy occurred at least 2.5 months before the birth of those offspring (data not shown).

## Discussion

Within the current epidemiologic context of pertussis, many countries in which the number of cases have increased in mainly newborns and infants not receiving primary doses because of age (Fabricius et al., 2018; Agrawal et al., 2019) have once again begun discussion on neonatal vaccination and its possible implementation (Lumbreras Areta et al., 2019). Neonatal immunization would provide an early protection for newborns and infants, thus narrowing the critical period of vulnerability intrinsic to routine vaccination schedules that start later in life. This strategy is easily implementable—given that birth is a crucial point of contact with the healthcare systems globally—and could also cover the gaps that the recently implemented maternal vaccination leaves, either because of the low coverage achieved in pregnant women or as a result of preterm birth. This strategy has, however, potential limitations that include poor immunogenicity (PrabhuDas et al., 2011), an immune response skewed toward a Th2-dominated immunity (Dowling and Levy, 2014), safety concerns, and the development of hyporesponsiveness to the same antigen and/or concomitant antigens administered at birth or in the subsequent months (Siegrist, 2003; Niewiesk, 2014). Nevertheless, the effectiveness of the currently used vaccines in the neonatal period is proof of the concept that vaccines can be successfully administered to the newborns to induce protection against different diseases (Wood and Siegrist, 2011).

In the study reported here, we used a mouse model to analyze whether neonatal immunization with pertussis vaccines that induce different Th-profiles—an OMVP-based vaccine giving rise to a Th1/Th2/Th17 mixed response, the aP resulting in mainly Th2, and the wP inducing Th1/Th17—led to a protection of offspring against *B. pertussis*. In agreement with previous findings on the preferential differentiation of CD4^+^ helper T cells into Th2 cells in response to danger signals and antigens in neonates (Mohr and Siegrist, 2016), the protection against *B. pertussis* was higher in neonatal mice immunized with 1 dose of the vaccine inducing mainly Th2 (aP) than the coverage recorded after immunization with the OMVP or wP vaccines. The protection against pertussis—as evidenced in a significant reduction in the bacterial colonization of the lungs in the mice challenged with sublethal doses of *B. pertussis* (Figure 3)—was increased in the schemes consisting of two immunizations, with a first dose at 7 days and a second at 21 days. In these two-dose schedules, the increase occurred to the greatest extent both when the aP vaccine was used for the two doses and when mixed schemes were implemented that contained aP for the first dose and OMVP or wP for the second. The scheme consisting of the OMVP-based vaccine in the first dose and the aP in the second also proved to be successful in terms of causing a reduction in the CFUs in comparison to non-immunized mice by more than 2 orders of magnitude (*p* < 0.001). We need to stress here that the two-dose schedules involving exclusively vaccines that induced immune profiles with Th1, such as wP or the OMVP-based vaccine, were not significantly effective (Figure 1B). This failure occurs because the neonatal immunologic milieu is polarized toward the Th2-type immunity with a dampening of the Th1-type responses (Zaghouani et al., 2009; Basha et al., 2014). The lower IL-12 production by dendritic cells was proposed as part of the cause of the lower capability of neonatal cells to generate memory cells and Th1-effector responses (Zhang et al., 2017). Furthermore, neonates do not yet have an anatomical microenvironment that is fully developed and thus equipped for the interaction between dendritic cells and T and B lymphocytes (Marchant and Kollmann, 2015). In fact, no defined demarcation exists between different lymphoid zones and T-CD4 zones in neonates. In agreement with that information, we detected no *B. pertussis–*specific T cells in the mice that only received one dose at 7 days of age (data not shown), suggesting that protection was mediated by anti(*B. pertussis*) antibodies rather than by cell-mediated immunity. The enhanced levels of protection detected for the aP-aP schedules, and for those that included neonatal immunization with the aP vaccine, was paralleled by increased levels of anti-PTx antibodies. Of interest to us was that the antibodies generated in mice from mixed-immunization schedules were of higher avidity than those elicited in the aP-aP–immunized mice and especially that, for the mixed-vaccination schemes, the increase in the percentage of elution of anti-PTx antibodies in the presence of NH_4_SCN (for the lower binding avidity; Figure 4A, second column) was always greater after a neonatal dose of aP, thus suggesting the presence of a prime-boost effect. Other evidence of that effect was the highest IgG2a/IgG1 ratios detected for mixed schemes having a neonatal dose with vaccines that induced a Th1 profile, that is OMVP or wP (Figure 4A, sixth column). Consistent with the IgG titers and the results of the avidity assays, we observed a differential recognition intensity of PTx in blots from SDS-PAGE gels probed with the vaccine-induced sera (Figure 4B). These results demonstrate the necessity of choosing the appropriate type of vaccine to be used in an immunization schedule beginning with a neonatal dose. Moreover, an evaluation of the type of adjuvant to use is essential. Although alum is the one most widely used, others are being tested to ensure an adequate response in the neonates. The capability of the oil-in-water emulsion called MF59 as an adjuvant in early life has begun to be investigated (Morris and Surendran, 2016). This adjuvant, when used for neonatal immunization, was recently found to induce a prolonged stimulation of a vaccine antigen and to enhance a recruitment of antigen-presenting cells and subsequently a promotion of CD4^+^-effector–T-cell activity (Morris and Surendran, 2016). Another approach that has been successfully tested in the murine model for promoting a robust T-cell response is the coadministration of IL-12 and the specific subunit vaccine to newborn mice. This combination accordingly led to an increased splenic expression of IFNγ and an enhanced protective efficacy of the tested vaccines (Arulanandam et al., 2000).

In our experiments, we also observed that in mice with maternal aP antibodies, the protection level induced by the transferred maternal immunity was not affected by neonatal immunization performed with any of the vaccines tested (Figure 5B). Under these conditions—and in contrast to what was observed in vaccination schemes consisting of a single neonatal dose in mice without maternal immunity—appreciable levels of IgG against PTx were detected (Figure 6A). What is noteworthy, however, is that, in the example of neonatal immunization with the aP vaccine, the IgG levels against PTx were significantly lower than those detected in the sera of the mice immunized at 7 days with the OMVP or wP vaccines, or those detected in the neonates with maternal immunity alone (*p* < 0.05 throughout). We also observed similar results in pups born to mothers whose immunization during pregnancy had occurred at least 2.5 months before the birth of those offspring (data not shown). This phenomenon, known as blunting, was confirmed by immunoblotting assays (Figure 6B; the second blot). The results obtained by these assays demonstrated that the recognition against PTx was null for the sera from aP-vaccinated offspring born of mothers that had been immunized with that same vaccine during pregnancy. As in naïve animals, the antibodies induced by that aP neonatal dose were of lower avidity than those raised by the OMVP or wP vaccines.

The mechanism by which maternal antibodies limit the offspring’s vaccine responses is still poorly understood. Vono et al. (2019) evidenced that maternal immunity exerts an influence on the germinal-center–B-cell differentiation into antibody-secreting plasma cells. Those authors proposed that high-avidity maternal antibodies bind to immunodominant epitopes and are thereafter able to force the binding of early-life B cells into non-immunodominant epitopes of lower affinity (Vono et al., 2019). This mechanism would then limit their chance of surviving through the germinal-center selection and plasma-cell differentiation (Phan et al., 2006). The authors speculated that the use of different vaccines in mothers and infants was possibly recruiting disparate types of B cells into the immunodominant response. This hypothesis is supported in the present work by the observation that antibody responses generated in aP-vaccinated offspring born to aP-immunized mothers was of lower avidity than in both the OMVP- or wP-vaccinated Ipups.

In the animals born at least 2.5 months after their mothers received the last aP dose and then were immunized with a neonatal dose of aP, a slight decrease in protection was observed in parallel to the IgG titers and the avidity results. We need to stress, however, that the levels of protection detected for the Ipups with maternal immunity born 3 weeks after their mothers received the last aP dose and then vaccinated at 7 days with aP were similar to those registered with the pups from mothers either unvaccinated or vaccinated with the other two vaccines tested. The differences in protection detected between the neonates immunized with aP and born at 3 weeks and the offspring immunized with aP but born at 2.5 months after maternal immunization could be due to differences in the proportion of maternal antibodies that mediate an effective protection in pups, with those being at a higher titer in pups born at 3 weeks after the last maternal booster. The explanation could be that in mice born after maternal immunization, high-avidity maternal antibodies bind to immunodominant epitopes present in the vaccine used for neonatal immunization (causing the blunting effect); but apart from that occurrence, other maternal antibodies capable of inducing a robust protective response are still present. The levels of those antibodies would be dependent on the time of birth after the last maternal immunization. Unfortunately, because of the design of our experiment, this hypothesis could not be tested since the discrimination of maternal antibodies from those induced by neonatal vaccination is still not possible.

As expected from previous reports on the aP vaccine (Allen et al., 2018), we detected in preliminary assays that in the animals born either at 3 weeks or at least 2.5 months after their mothers received the last aP dose, the aP neonatal dose did not prevent *B. pertussis* colonization in the nose.

A major finding from our work was that neonatal immunization with OMVP or wP at any of the times tested (3 weeks or 2.5 months after maternal immunization) did not affect the protection conferred by maternal immunity.

The present results on the dependence of interference in immunization efficacy by the types of vaccine used for the mothers and the infants are in agreement with those reported by Feunou et al. (2016), who demonstrated that the protection induced by neonatal vaccination was affected by maternal antibodies when the vaccine used for the neonatal dose was the same as that used in the pregnancy. When the mothers and the infants were immunized with two different types of vaccines, no interference of neonatal vaccination in the protective effects of maternal antibodies was noted.

All the results presented here demonstrate that in this murine model, the application of a neonatal-vaccination strategy against pertussis is feasible even in those infants who have maternal immunity. For infants with maternal immunity induced by the aP vaccine, however, using a second type of vaccine for neonatal immunization and/or a different adjuvant would be advisable (Sakala et al., 2019). Though the use of mouse models to investigate neonatal vaccination is admittedly not a forum for completely replicating human physiology, the results obtained with a model of this design will nevertheless enable a test of the proposed hypotheses under controlled conditions, where the forthcoming results can then refine those concepts for further validation in subsequent human studies.

## Data Availability Statement

All datasets generated for this study are included in the article/supplementary material.

## Ethics Statement

The animal study was reviewed and approved by the Ethical Committee for Animal Experiments of the Faculty of Science at La Plata National University (approval number 004-06-15, 003-06-15 extended its validity until August 10, 2023).

## Author Contributions

DH planned the study, performed the laboratory analyses, interpreted the data, and drafted the manuscript. DB planned the study, interpreted the data, and revised the figures and the manuscript. MG and MZ performed certain experiments, interpreted the data, and revised the figures and the manuscript. PM and NA performed the experiments and the laboratory analyses. All the authors approved the final version of the manuscript.

## Conflict of Interest

DH is a member of the “Global Pertussis Initiative” (supported by Sanofi Pasteur, United States). The remaining authors declare that the research was conducted in the absence of any commercial or financial relationships that could be construed as a potential conflict of interest.
